# Multiscale Analysis of the Impact of the Isomerization of 4‑Amine Tetraortho-Azobenzenes on the Structure and Dynamics of Tubular Micelles

**DOI:** 10.1021/acs.langmuir.6c02376

**Published:** 2026-06-18

**Authors:** Alberto S. Luviano, Natalia Rincón-Londoño, Sandra Ramírez-Rave, Jesús Gracia-Mora, Maria Josefa Bernad-Bernad, Anatoly K. Yatsimirsky

**Affiliations:** † Departamento de Ingenierías Química, Electrónica y Biomédica, División de Ciencias e Ingeniería, 197573 Universidad de Guanajuato, Campus León, León, Guanajuato 37150, México; ‡ Departamento de Ingeniería Física, División de Ciencias e Ingenierías, Universidad de Guanajuato, León, Guanajuato 37150, México; § Facultad de Química, 164178 Universidad Nacional Autónoma de México, Ciudad Universitaria, Coyoacán, Ciudad de México 04510, México

## Abstract

A multiscale analysis of photoresponsive micellar systems based on CTAB/NaSal and 4-amine tetraortho-azobenzenes (AzoCH_3_ and AzoCl) was carried out to elucidate how molecular photoisomerization modulates micellar morphology, mesoscopic organization, and macroscopic dynamics in the semidilute regime. Under basic conditions (pH 11), CTAB/NaSal solutions incorporating trans-AzoCH_3_ and trans-AzoCl form well-defined wormlike micelles (WLMs), as confirmed by TEM and molecular dynamics simulations, which reveal the presence of tubular structures. Dynamic light scattering (DLS) measurements indicate that these systems lie within the semidilute–entangled regime, where collective hydrodynamic behavior dominates over individual micellar dimensions, in agreement with viscosity measurements. Upon UV irradiation at 360 nm, *trans–cis* photoisomerization of the azobenzenes induces a pronounced disruption of the WLM network, leading to partial collapse and fragmentation of tubular structures, as evidenced by HR-TEM imaging and simulations. Despite this significant structural rearrangement, DLS measurements reveal no substantial change in the hydrodynamic correlation length, indicating that the system remains in the semidilute regime, consistent with the slight decrease in viscosity while preserving shear-thinning behavior. Thermal *cis*-to-*trans* isomerization kinetics were monitored by UV–vis spectroscopy and were well described by monoexponential behavior, confirming reversible photosensitivity within the micellar environment. These results demonstrate that azobenzene photoisomerization can modulate micellar morphology without altering the overall concentration regime, highlighting a decoupling between local micellar structure and mesoscopic collective dynamics. This behavior underscores the robustness of semidilute wormlike micellar networks and their potential for applications in light-responsive soft materials.

## Graphical abstract

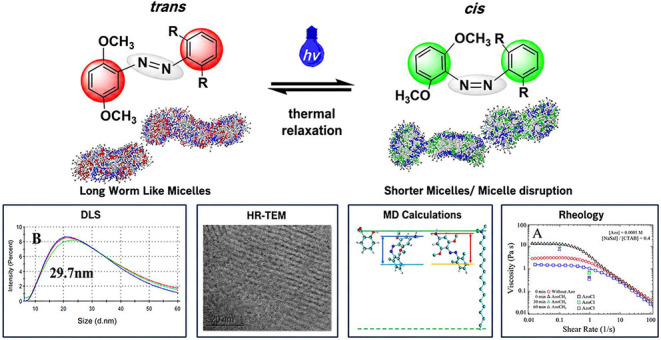

## Introduction

Surfactant self-assembly in aqueous media gives rise to a wide variety of micellar structures whose morphology and dynamics are highly sensitive to concentration, ionic strength, and the presence of additives.^1–3^ Among these systems, cationic surfactants such as cetyltrimethylammonium bromide (CTAB) have been extensively studied due to their ability to form spherical, cylindrical, and wormlike micelles upon variation of solution conditions.^4,5^ In particular, the addition of aromatic counterions such as sodium salicylate (NaSal) promotes micellar growth by screening electrostatic repulsion between headgroups, leading to the formation of long, flexible wormlike micelles (WLMs) that exhibit pronounced viscoelastic behavior.^6,7^

Wormlike micellar solutions typically exist in the semidilute regime, where micelles overlap and form transient networks characterized by collective hydrodynamic correlations rather than isolated particle dynamics.^8^ In this regime, techniques such as dynamic light scattering (DLS) probe mesoscopic correlation lengths associated with micellar interactions, while viscosity measurements reflect the macroscopic response of the tubular micellar network under applied deformation. In contrast, transmission electron microscopy (TEM) provides direct visualization of local micellar morphology,^9^ and molecular dynamics simulations provide complementary representations of the microscopic structural configurations, enabling visualization of the system morphology and calculation of the density number distribution.^10^ Importantly, changes in micellar structure do not necessarily imply changes in the concentration regime, highlighting the need to distinguish between local aggregate geometry and collective solution behavior.^11^

The incorporation of photoresponsive molecules into micellar systems offers an effective strategy for externally controlling self-assembly and material properties.^12^ Azobenzene derivatives are particularly attractive in this context due to their reversible *trans–cis* photoisomerization, which induces substantial changes in molecular geometry, polarity, and packing constraints.^13–15^ When embedded within micellar assemblies, azobenzenes can disrupt micellar curvature and stability, potentially triggering structural transitions upon light irradiation.^16–18^ Despite extensive studies on photoresponsive surfactant systems, the relationship between azobenzene isomerization, micellar morphology, and concentration regime in semidilute wormlike micellar networks remains poorly understood.

The azobenzene derivatives selected for this study belong to a family of tetra-ortho-substituted 4-aminoazobenzenes previously investigated by our group. AzoCH_3_ and AzoCl were chosen because they possess similar molecular dimensions while differing in the electronic nature of the substituent attached to the azo-containing aromatic ring. The methyl group exhibits electron-donating character, whereas chlorine exerts an overall electron-withdrawing effect, leading to differences in polarity, acid–base properties, and intermolecular interactions. These characteristics make both compounds suitable molecular probes for evaluating how subtle electronic modifications influence the interaction between azobenzenes and wormlike micellar environments. The CTAB/NaSal composition employed in this work was deliberately selected above the critical shape transition concentration (CSTC) in order to ensure the formation of a stable wormlike micellar network. This approach allows the effects of azobenzene incorporation and photoisomerization on an already established semidilute wormlike micellar system to be isolated from structural fluctuations associated with the sphere-to-wormlike transition itself.

In this work, we investigate the structural, dynamic, and kinetic consequences of incorporating azobenzene derivatives (AzoCH_3_ and AzoCl) into CTAB/NaSal micellar systems under basic pH conditions. Using a combination of DLS, viscosity, HR-TEM, molecular dynamics simulations, and UV–vis spectroscopy, we examine how photoinduced trans–cis isomerization affects micellar morphology, while assessing whether the semidilute regime is preserved. In addition, the kinetics of thermal *cis*-to-*trans* relaxation within the tubular micellar environment are quantified spectrophotometrically. Our results demonstrate that photoisomerization induces significant morphological rearrangements of wormlike micelles without disrupting the underlying semidilute concentration regime, providing insight into the decoupling of local structure and collective dynamics in photoresponsive soft-matter systems.

## Materials and Methods

### Materials

4-amine tetraortho-azobenzenes AzoCH_3_ and AzoCl were synthesized according to the protocol published by Wang et al.^19^ Cetyltrimethylammonium bromide (CTAB, purity >99%), sodium salicylate (NaSal, purity >99.5%), and sodium carbonate (Na_2_CO_3_, purity >99.5%) were purchased from Sigma-Aldrich. Sodium hydroxide (NaOH, purity >95%) was obtained from CTR Scientific. All chemicals were used without further purification. Ultrapure water with a resistivity of 18.2 MΩ·cm was obtained from a Millipore system. Unless otherwise specified, the CTAB/NaSal systems discussed throughout this work correspond to solutions containing 0.05 M CTAB and 0.02 M NaSal ([NaSal]/[CTAB] = 0.4). Therefore, whenever concentrations are not explicitly reported, this composition should be assumed.

### Kinetic Measurements

The kinetics of the thermal *cis*-to-*trans* isomerization of the azobenzenes AzoCH_3_ and AzoCl embedded in CTAB/NaSal tubular micelles were investigated by UV–vis spectrophotometry. Solutions containing 5 × 10^–5^ M of the corresponding azobenzene were prepared in distilled water with CTAB (0.05 M) and sodium salicylate (0.02 M). The pH was adjusted to 11 using 0.05 M Na_2_CO_3_ as a buffer in order to suppress the catalytic effects associated with acidic impurities.^30^ The resulting solutions were transferred to quartz cuvettes and placed in the thermostated cell holder of a spectrophotometer equipped with a Hewlett–Packard 89090A Peltier temperature controller. After thermal equilibration at 25 °C, the samples were irradiated directly inside the spectrophotometer compartment using a 9 W UV lamp (λ = 360 nm) to induce *trans*-to-*cis* photoisomerization of the azobenzene moiety. UV–vis absorption spectra were recorded at approximately 10 s intervals to monitor the photoisomerization process. Once the photostationary state (PSS) was reached (typically within 2–3 min) the irradiation was discontinued, and the subsequent thermal cis-to-trans isomerization was followed by recording UV–vis spectra at appropriate time intervals, depending on the reaction rate. The resulting absorbance–time profiles were fitted to a monoexponential kinetic model using nonlinear least-squares regression implemented in Origin 9.1

### DLS Measurements

The hydrodynamic diameter was determined by dynamic light scattering (DLS) in a Zetasizer Nano ZS using glass and dip cells for measurements. A Zetasizer Nano-ZS 4800 (Malvern Instruments, U.K.) was used for DLS experiments. The light source was an Ar-ion laser operating at a wavelength of 633 nm at 175° scattering angle. Each sample was filtered by 0.45 μm filter before use. Stokes–Einstein relationship was used to calculate the apparent hydrodynamic diameter (*d*_H_) of the micelles from cumulant analysis. Resultant micelles were dispersed in deionized water (5 × 10^–5^ M of the corresponding azobenzene with CTAB 0.05 M and sodium salicylate 0.02 M at pH 11, employing 0.05 M Na_2_CO_3_ as a buffer) and sonicated for 10 min. The measurements were performed at 25 °C.

### HR-TEM

High resolution transmission electron microscopy (HR-TEM) analyses were performed using a JEMARM200F instrument (JEOL Japan) operating at 200 kV. Resultant micrographs were analyzed employing the software ImageJ.

### Viscosity Measurements

Viscosity measurements were performed over a range of shear rates using a stress-controlled rheometer (DHR-3, TA Instruments) equipped with a cone–plate geometry (40 mm diameter, 0.5081° cone angle). All measurements were conducted at 25 °C using a Peltier temperature control system, and a solvent trap was employed to prevent water evaporation.

The samples were prepared by first dispersing AzoCH_3_ or AzoCl in water at concentrations of 5 × 10^–4^ and 0.01 M. Subsequently, CTAB (0.05 M) and sodium salicylate (0.02 and 0.05 M) were added to each dispersion at pH 11, using 0.05 M Na_2_CO_3_ as a buffer. The samples were measured over a range of shear rates.

Aliquots of 2 mL of each sample were irradiated using a commercial UV lamp with a power output of 11.8 mW at 360 nm for 30 and 60 min prior to viscosity measurements, which were carried out in darkness. During irradiation, the samples were positioned 10 cm from the lamp, and temperature was controlled using a Peltier system to avoid thermal fluctuations. After irradiation, the viscosity was measured at a fixed shear rate for 2 min, since measurements over a range of shear rates require approximately 10 min. Viscosity measurements were performed by triplicate.

### Molecular Dynamics Simulations

Molecular dynamics (MD) simulations were performed using the GROMACS 2019 software package, employing the GROMOS 54a7 force field to model interatomic interactions.^20,21^ Prior to production, all systems underwent energy minimization using the steepest descent algorithm, followed by equilibration under NVT and subsequently NPT conditions for 2 ns each. Production trajectories were generated in the NPT ensemble. Temperature was maintained at 25 °C using the V-rescale thermostat, while pressure was controlled at 1 bar with a compressibility of 4.5 × 10^–5^ bar^–1^ using the Parrinello–Rahman barostat.^22,23^ Electrostatic interactions were computed using the particle mesh Ewald (PME) method with a real-space cutoff of 1.2 nm.^24,25^ All-bond constraints were applied through the LINCS algorithm.^26^

Using the Automated Topolgy Builder (ATB) server,^27^ we generate the molecules of CTAB (CTAB+), NaSal (Sal-) and the AzoCH_3_ and AzoCl *trans*- and *cis*- isomers. The topologies of both Azo molecules were constructed using the crystallographic results of the molecules in *trans* configuration, *cis* isomers were estimated by calculations. Ions were added from the ions bank of the GROMOS 54a7 force field. The simple point/charge extended (SPC/E) water model was used to solvate all the simulated systems.^28^

To obtain the desired simulation systems, an initial box of 4.5 × 4.5 × 36 nm^3^ was constructed, containing 700 CTAB, 280 NaSal and 140 *trans*-Azo (or *cis*-Azo) molecules randomly distributed. The box was expanded to a 10 × 10 × 40 nm^3^ box, followed by solvation and the addition of ions. The same procedure was applied for AzoCH_3_ and AzoCl systems.

## Results and Discussion

Azobenzenes studied in this work are displayed in Figure 1. The spectrophotometric evolution of the *cis–trans* isomerization reaction is shown in Figure 2. The characteristic absorption bands of azobenzenes, corresponding to the π–π* and n−π* transitions, are clearly observed. However, when compared with the spectra of both compounds recorded in water, these bands exhibit noticeable shifts, which can be attributed to changes in polarity associated with the micellar environment. For the *trans* isomers, a bathochromic shift of the π–π* band is observed in both cases. For AzoCH_3_, this band appears at approximately 355 nm in water under basic pH conditions, whereas in the CTAB/NaSal micellar system it is shifted to 360 nm. A similar behavior is observed for the n−π* band: while it absorbs at around 447 nm in water, it is red-shifted to 460 nm in the CTAB/NaSal tubular micelles. AzoCl follows the same trend, with absorption maxima at 367 and 450 nm in water, which shift to 380 and 460 nm, respectively, in the CTAB/NaSal micellar medium. The wavelength values employed to compare the ones obtained in this work were taken by a pervious report already published in the literature.^29^

**Figure 1:**
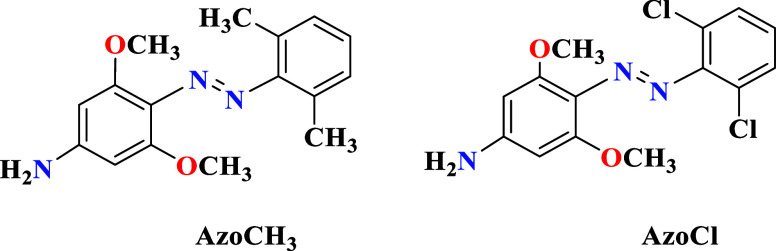
Structure of 4-amine tetraortho-azobenzenes tested in the current work.

**Figure 2:**
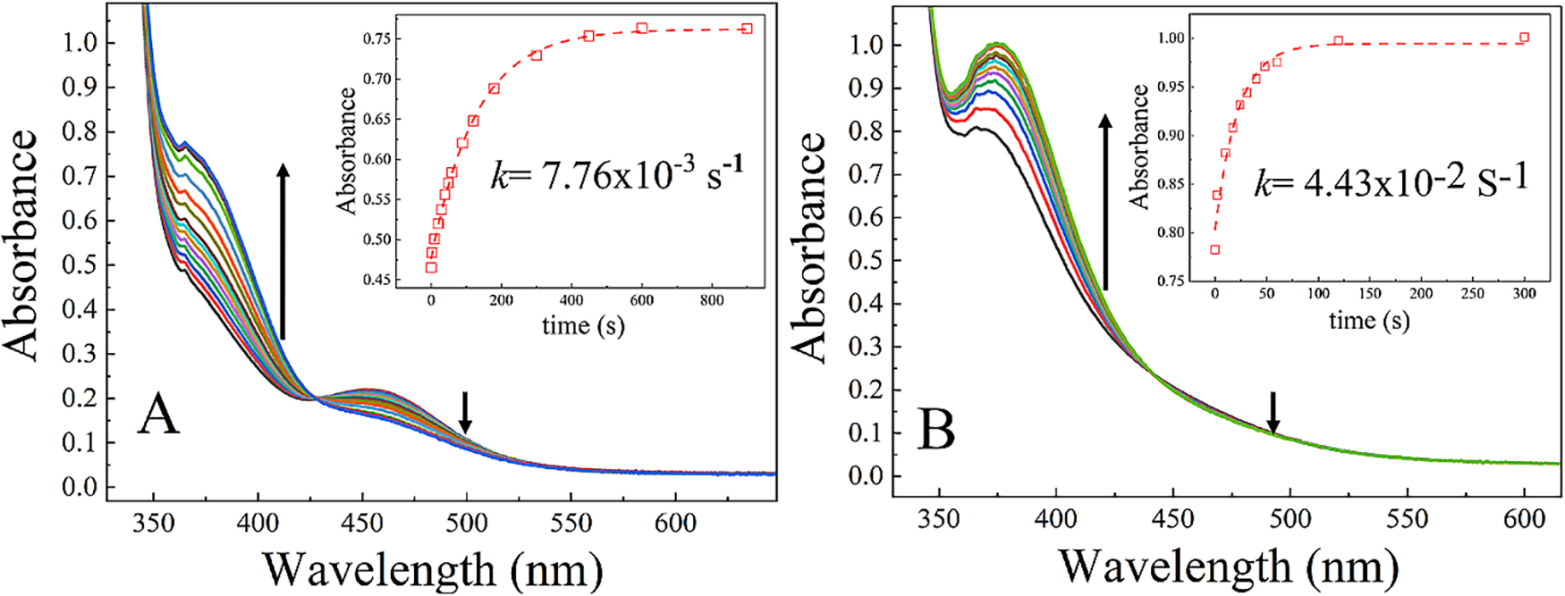
Spectrophotometric evolution of *cis* to *trans* isomerization reaction for (A) AzoCH_3_ and (B) AzoCl in CTAB/NaSal system at pH11. Insets show absorbance vs. time profiles and their fits (solid lines) to the first-order kinetics. For AzoCl, the *n*–π* transition appears significantly less intense than in AzoCH_3_. This behavior is attributed to the electronic effect of the chlorine substituent, which modifies the relative intensities of the electronic transitions and leads to partial overlap of the *n*–π* band with the tail of the more intense π–π* transition. Nevertheless, the band remains observable and follows the expected evolution during the photoisomerization process.

Irradiation of solutions containing both azobenzenes embedded in the micellar systems at a λ of 360 nm, leads to the formation of the corresponding photostationary state (PSS). Under these conditions, the π–π* band decreases markedly, while the n−π* band shows a slight increase. In addition, the appearance of a single isosbestic point (at 425 nm for AzoCH_3_ and 445 nm for AzoCl) confirms the coexistence of two interconverting species, namely the *trans* and *cis* isomers. These spectral changes are consistent with a typical *trans*-to-*cis* photoisomerization process in azobenzenes.^29,30^

Figure 2A,B include insets showing the monoexponential fits used to extract the first-order rate constants for the thermal *cis*-to-*trans* isomerization (*k*_c→t_). These constants were obtained from spectra recorded during the thermal relaxation process in the dark at 25 °C. The values determined for AzoCH_3_ (7.76 × 10^–3^ s^–1^) and AzoCl (4.43 × 10^–2^ s^–1^) in CTAB/NaSal micelles are significantly lower than those measured in water (0.079 s^–1^ for AzoCH_3_, while for AzoCl the reaction was too fast to be detected at basic pH). However, they are comparable to those reported in *n*-butanol in the same study (7.40 × 10^–3^ s^–1^ for AzoCH_3_ and 2.40 × 10^–2^ s^–1^ for AzoCl).^30^ As is well-known, azobenzenes act as sensitive probes of their local environment, and the *k*_c→t_ value serves as an indicator of the medium in which the isomerization occurs. Therefore, the similarity between the *k*_c→t_ values obtained in CTAB/NaSal micelles and in n-butanol suggests that both azobenzenes reside in a polar region of the micelles. This region is less polar than bulk water but still significantly polar, allowing one to infer that AzoCH_3_ and AzoCl are preferentially located near the Stern layer rather than in the nonpolar micellar core. These results are consistent with the localization obtained from the simulations, which will be discussed in detail later. Notably, the *k*_c→t_ value for AzoCH_3_ in the CTAB/NaSal system remains unchanged when the isomerization experiment is repeated using the same solution after 1, 3, and 7 days, indicating good temporal stability of the system. Spectra recorded in the repetition experiment at 7 days is displayed in Figure SI1. The observation of thermal *cis→trans* relaxation after removal of the irradiation source constitutes direct evidence of the reversibility of the photoisomerization process within the CTAB/NaSal micellar environment. The recovery of the characteristic spectral features of the *trans* isomer and the successful monoexponential fitting of the absorbance–time profiles demonstrate that the photoswitching process remains reversible under the experimental conditions employed.

The supramolecular complex formed between AzoCH_3_ and CTAB exhibits the characteristic absorption bands of azobenzenes, with the π–π* transition centered at 365 nm and the n−π* transition at 455 nm (Figure SI2). When a solution of this complex (5 × 10^–5^ M AzoCH_3_ and 0.05 M CTAB) is titrated at pH 7 with a concentrated NaSal solution (0.2 M), several significant spectral changes are observed. Specifically, the π–π* band decreases progressively in intensity, while an intense absorption band associated with the salicylate anion, centered at approximately 300 nm,^31^ emerges and grows markedly as the NaSal concentration increases. In contrast, the band at 455 nm increases. These spectral changes indicate that the azobenzene undergoes a transformation from its neutral form to its protonated form within the micellar environment as a consequence of the effect of salicylate on the net charge of the system. In aqueous solution, this azobenzene exhibits a reported p*K*_a_ of approximately 7;^29^ however, upon incorporation into positively charged CTAB micelles, a significant decrease in the effective p*K*_a_ is expected due to electrostatic interactions.^32^ Upon addition of a counterion such as salicylate, charge screening becomes apparent not only through the structural transition of the micelles from spherical to tubular aggregates,^33,34^ but also through concomitant changes in the physicochemical properties of the embedded dye, including its acid–base equilibrium (raise of p*K*_a_).

Under these conditions, the azobenzene displays the coexistence of its neutral and protonated forms, as evidenced by the presence of two well-defined isosbestic points at 350 and 420 nm. At low salicylate concentrations (below 0.015 M), the effect of the counterion on the optical properties of azobenzene is negligible. In contrast, at higher salicylate concentrations, pronounced spectral changes are observed, and the formation of the protonated AzoCH_3_ form becomes evident, consistent with previously reported acid–base equilibria for AzoCH_3_.^29^

As shown in Figure 3, the addition of NaSal induces a transition from spherical micelles to wormlike micelles. Notably, the onset of these spectral changes occurs at a NaSal concentration of approximately 0.015 M, corresponding to a NaSal/CTAB molar ratio of 0.30. This concentration is therefore assigned as the critical shape transition concentration (CSTC). This value was independently corroborated by dynamic light scattering (DLS) measurements carried out during an analogous titration experiment, which reveal a clear transition from spherical micelles to wormlike micelles (WLMs) at a NaSal concentration comparable to that identified by spectroscopic analysis. This molar ratio for CSTC is consistent with other reports based in CTAB/NaSal WLM published before.^35^

**Figure 3:**
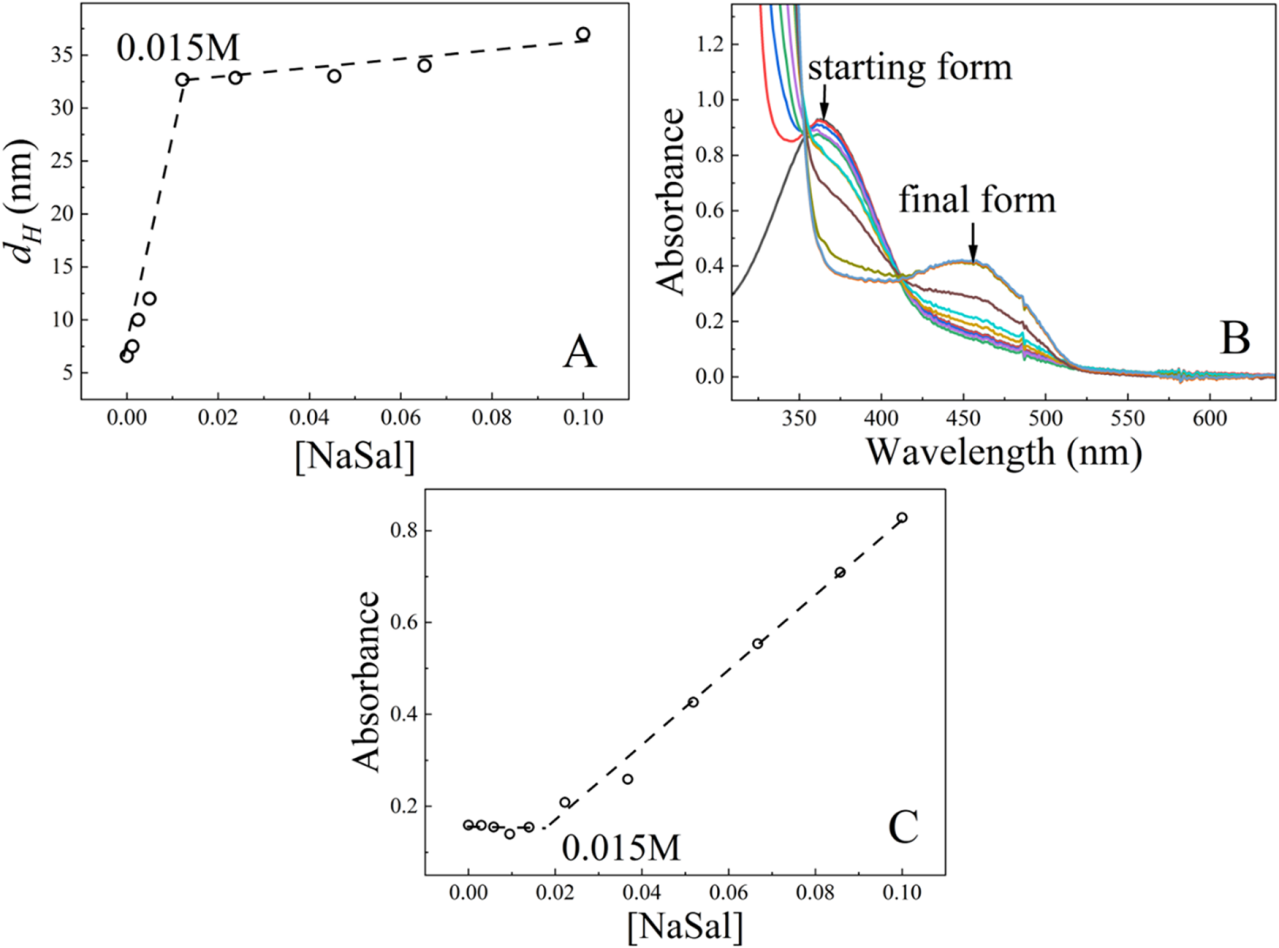
Structure dependence on the NaSal concentration in AzoCH_3_ CTAB/NaSal system followed by (A) DLS, (B) and (C) UV–vis spectroscopy at pH 7.

Samples of CTAB at a concentration of 0.05 M were analyzed through DLS both in the absence and in the presence of the two azobenzenes, as shown in Figure 4. These measurements indicate that CTAB micelles at this concentration form spherical aggregates with an average diameter of approximately 6 nm, as illustrated in inset 4 (A). Upon incorporation of AzoCH_3_ or AzoCl, only a negligible increase in micellar size is observed. Specifically, the diameters increase to 6.21 nm for AzoCH_3_ and 6.10 nm for AzoCl, indicating that loading with either azobenzene does not significantly perturb the micellar structure. Samples with other concentrations of both azobenzenes (2.5 × 10^–5^, 5 × 10^–4^ and 5 × 10^–3^ M) were also recorded but no significant changes were observed. Although only minor changes in hydrodynamic diameter are observed, these results should not be interpreted as evidence of the absence of azobenzene incorporation. Because the azobenzene concentration (5 × 10^–5^ M) is approximately 3 orders of magnitude lower than the CTAB concentration (0.05 M), only a small fraction of surfactant molecules interacts directly with the dye molecules. Under these conditions, large changes in micellar dimensions are not expected. Nevertheless, the significant changes observed in UV–Vis spectra, thermal isomerization kinetics, and molecular dynamics simulations (Figure SI3) demonstrate that both azobenzenes interact strongly with the micellar environment.

**Figure 4:**
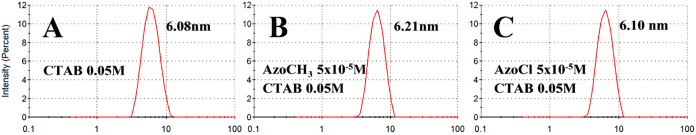
DLS experiments recorded for (A) CTAB 0.05M, (B) AzoCH_3_ 5 × 10^–5^ M/CTAB 0.05 M and (C) AzoCl 5 × 10^–5^ M/CTAB 0.05 M at pH = 11.

Figure 5 displays the corresponding DLS experiments carried out for CTAB/NaSal systems. At a fixed composition of 0.05 M CTAB and 0.02 M NaSal, the resulting wormlike micellar solutions fall within the semidilute-entangled regime, in which the characteristic length scale probed by dynamic light scattering (DLS) reflects a collective relaxation mode associated with hydrodynamic correlations within the micellar network rather than the geometric dimensions of individual micelles. In this regime, the hydrodynamic diameter (*d*_H_) obtained from DLS is related to the hydrodynamic correlation length (ξ_H_) through the scaling relation *d*_H_ ≈ 2ξ_H_, such that ξ_H_ = *d*_H_/2.

**Figure 5:**
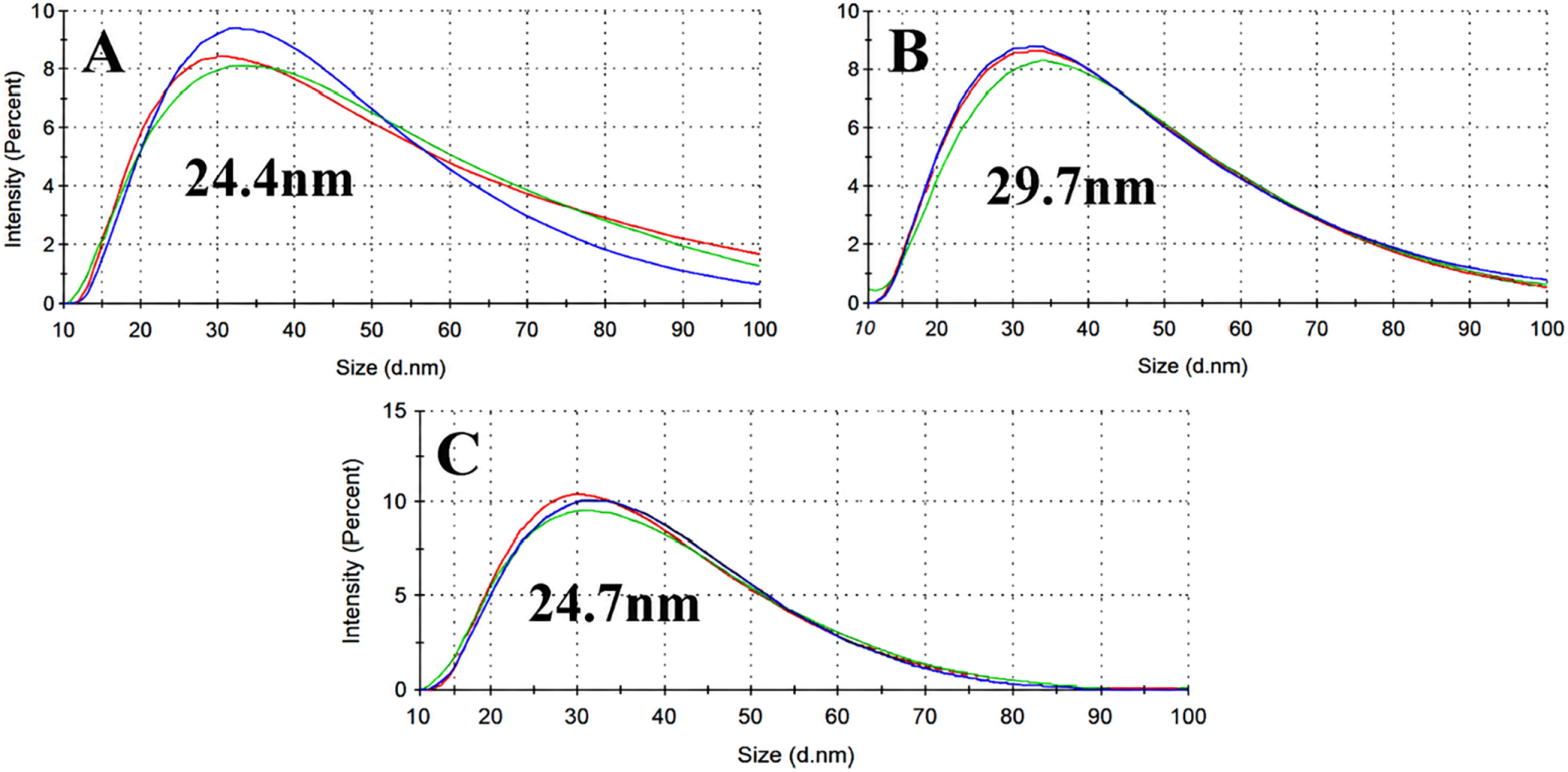
DLS experiments recorded for (A) CTAB/NaSal, (B) AzoCH_3_ CTAB/NaSal and (C) AzoCl CTAB/NaSal systems at pH = 11.

For the dye-free reference system, a hydrodynamic diameter of *d*_H_ = 24.4 nm is obtained, corresponding to ξ_H_ = 12.2 nm. Upon incorporation of AzoCH3 at a concentration of 5 × 10^–5^ M, *d*_H_ increases to 29.7 nm, yielding ξ_H_ = 14.9 nm (see Figure SI5), whereas the addition of AzoCl at the same concentration results in a hydrodynamic diameter of 24.7 nm (ξ_H_ = 12.4 nm), essentially indistinguishable from the reference system (see Figure SI6). Considering that these measurements were performed at pH 11, the obtained values are in good agreement with those reported by Patel et al.^34^ for CTAB-based micellar systems incorporating different aromatic carboxylic acids as counterions.

The micellar volume fraction (ϕ) was estimated from the CTAB concentration, yielding ϕ ≈ 0.018. The structural mesh size (ξ) of the semidilute network was estimated using ξ ≈ *a*ϕ^–1/2^, where *a* is the tubular micelle radius. Using *a* ≈ 3 nm gives ξ ≈ 22 nm. It should be noted that ξ represents the network mesh size, whereas ξ_H_ corresponds to the hydrodynamic correlation length obtained from DLS. Although both parameters characterize the semidilute network, they originate from different physical descriptions and therefore should not be interpreted as identical quantities. These calculated values are fully consistent with the structural characteristics expected for a semidilute, entangled micellar regime.^8^

The ratio between the hydrodynamic and structural length scales, ξ_H_/ξ ≈ 0.5–0.7, falls within the expected range for semidilute wormlike micellar networks, confirming that the incorporation of azobenzenes does not alter the underlying network topology. Consequently, the increase in ξ_H_ observed upon addition of AzoCH_3_ reflects a slight modification of micellar dynamics, most likely arising from enhanced hydrodynamic coupling and local dynamic stiffening of the network. This behavior is consistent with the spectroscopic evidence indicating stronger interactions of AzoCH_3_ with the micellar interface above the CSTC. In contrast, AzoCl induces only minimal changes in *d*_H_ and ξ_H_ and therefore behaves largely as a hydrodynamic spectator, in agreement with its weaker perturbation of both micellar structure and local physicochemical environment. Parameter calculated for micellar resulting systems are summarized in Table 1.

**Table 1:** Microstructural Parameter of WLM Obtained in This Work in a Semidiluted Regime

system	*d*_H_ (nm)	ξ_H_ = *d*_H_/2 (nm)	ϕ	ξ (nm)	ξ_H_/ξ
CTAB/NaSal	24.4	12.2	0.018	22	0.55
AzoCH_3_ CTAB/NaSal	29.7	14.9	0.018	22	0.68
AzoCl CTAB/NaSal	24.7	12.4	0.018	22	0.56

Hydrodynamic diameters were measured for both systems with samples irradiated with a 360 nm lamp, and no significant variations were observed (As observed in Figure SI4), this result indicates that the photoisomerization process does not perturb the semidilute regime of the system. The absence of significant changes in hydrodynamic diameter after irradiation is consistent with the semidilute-entangled nature of the system. Under these conditions, DLS probes a collective hydrodynamic correlation length rather than the contour length of individual wormlike micelles. Consequently, shortening of the micellar contour length induced by photoisomerization may occur without producing measurable changes in the characteristic hydrodynamic diameter.

High-resolution transmission electron microscopy (HR-TEM) micrographs were acquired for the micellar systems under study. The samples were prepared under low-vacuum conditions and chemically fixed with phosphotungstic acid (PTA) to ensure adequate preservation of their structural features.^36^ Figure 6 shows an HR-TEM micrograph of the AzoCH_3_/CTAB system at pH 11, where well-defined spherical particles are clearly observed. These particles exhibit diameters in the range of 4.5–6.0 nm, which is consistent with the expected size of spherical CTAB micelles.^37^ Although some aggregates appear crowded in the micrograph, this behavior is attributed to staining, drying, and vacuum conditions during sample preparation, which promote close packing of the soft micellar structures on the TEM grid. Nevertheless, the spherical morphology remains clearly discernible. A representative spherical aggregate exhibits a diameter of approximately 5.6 nm, in excellent agreement with the hydrodynamic diameter obtained independently by DLS measurements (∼6 nm). As discussed throughout this work, TEM and DLS provide complementary information. Whereas TEM directly visualizes the local morphology of the aggregates, DLS probes their hydrodynamic behavior in solution. Therefore, the identification of spherical micelles is supported by the excellent agreement between both techniques.

**Figure 6:**
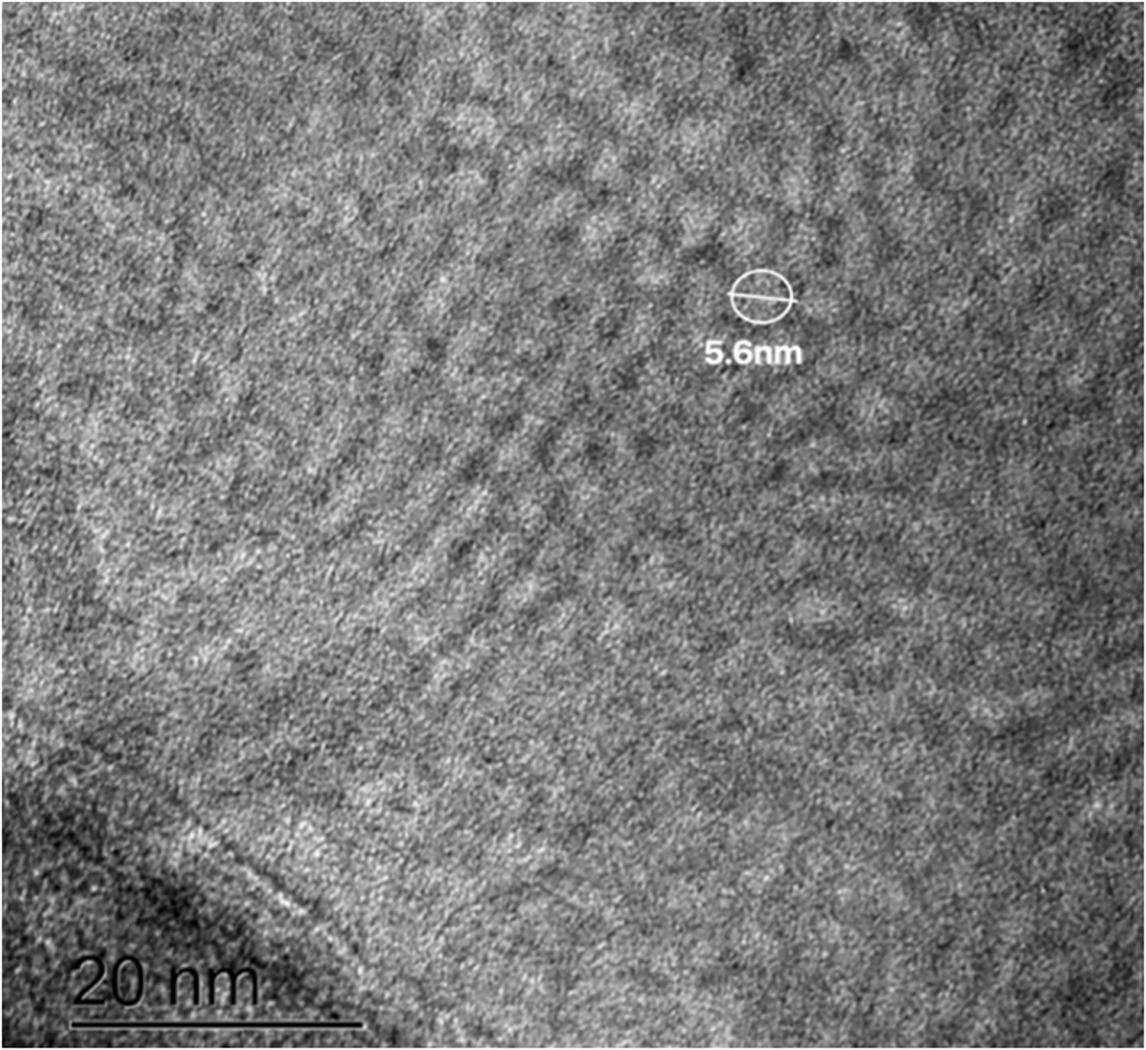
HR/TEM image of AzoCH_3_/CTAB spherical micelles at pH 11.

Figure 7A,B shows TEM micrographs acquired at different magnifications (20 and 100 nm), in which well-defined tubular structures are clearly observed. These structures correspond to the wormlike micellar (WLM) conformation expected for the AzoCH_3_ CTAB/NaSal system under basic pH conditions. The observed tube diameters are in the range of approximately 2.5–3.0 nm. Although the tubular morphology is well preserved, a slight shrinkage is evident, which can be attributed to the low-vacuum conditions employed during sample preparation.

**Figure 7:**
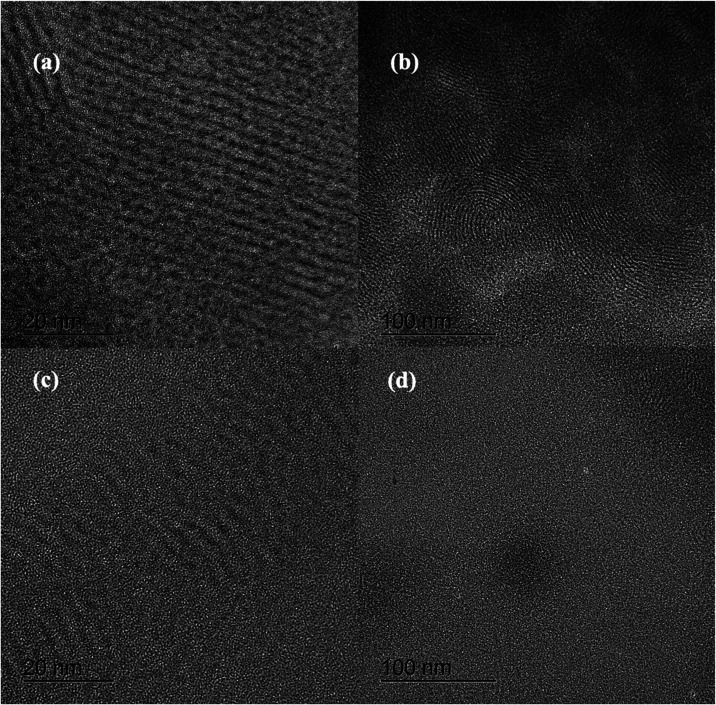
HR-TEM micrographs of the AzoCH_3_ CTAB/NaSal system at pH 11, (a) and (b) from the micelles with AzoCH_3_ in *trans* conformation, (c) and (d) in the *cis* form at different scales.

On the other hand, Figure 7C,D presents TEM images of the same AzoCH_3_ in the CTAB/NaSal system after irradiation at 360 nm to induce *trans–cis* isomerization of the AzoCH_3_ molecule, recorded at comparable length scales (20 and 100 nm). In this case, the tubular conformation is difficult observed at 100 nm, it seems that the WLM conformation appears collapsed. While a zoom at 20 nm is done tubular features are evidently discernible, indicating a significant disruption in the size of the tubes in the micellar architecture upon photoisomerization. Considering the reported results, although photoisomerization induces a marked change in micellar morphology, the system remains in the semidilute regime, as evidenced by the persistence of collective hydrodynamic behavior. It should be emphasized that the TEM observations are interpreted together with DLS measurements, rheological characterization, and molecular dynamics simulations. Therefore, the microscopy images should not be considered as standalone evidence but rather as part of a complementary multiscale characterization strategy. All techniques consistently indicate the presence of elongated wormlike micelles in the *trans* state and a reduction in contour length following photoisomerization.

Molecular dynamics simulations were used as a tool to study the molecular interactions and morphology of *trans*- and *cis*-AzoCH_3_ in aqueous CTAB/NaSal solutions, as well as *trans*- and *cis*-AzoCl in the same aqueous solution. Four independent simulations were performed to investigate the molecular arrangement: (i) *trans*-AzoCH_3_/CTAB/NaSal, (ii) *cis*-AzoCH_3_/CTAB/NaSal, (iii) *trans*-AzoCl/CTAB/NaSal, and (iv) *cis*-AzoCl/CTAB/NaSal.

Figure 8 shows snapshots of the four systems at the end of the simulations (200 ns). Visual inspection of Figure 8A,B reveals that the *trans*-AzoCH_3_/CTAB/NaSal system forms micelles with a tubular morphology, with Azo molecules preferentially located at the micellar surface. In contrast, the *cis*-AzoCH_3_/CTAB/NaSal system exhibits a slight shortening of the elongated tubular micelles, but again with the Azo molecules on the surface. This behavior has been previously reported in systems where Azo undergoes *trans–cis* photoisomerization, and in consistency with TEM images observed, which indicate a significant reduction in the length of the micellar tubes upon irradiation.^38,39^ Also, the surface localization of the Azo molecules in the micelle was obtained of analysis of the UV–vis spectra.

**Figure 8:**
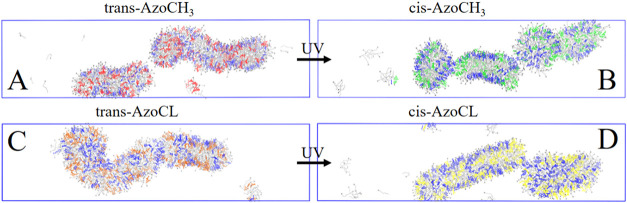
Molecular arrangement of the simulated systems, at 200 ns. (A) *trans*-AzoCH_3_/CTAB/NaSal, (B) *cis*-AzoCH_3_/CTAB/NaSal, (C) *trans*-AzoCl/CTAB/NaSal, and (D) *cis*-AzoCl/CTAB/NaSal. The color coding is the following, blue: NaSal, gray: CTAB, red: *trans*-AzoCH_3_, orange: *cis*-AzoCH_3_, green: *trans*-AzoCl and yellow: *cis*-AzoCl.

A rather similar behavior is observed for the AzoCl system (Figure 8C,D). The system containing *trans*-AzoCl exhibits a long tubular micellar morphology, while the *cis*-AzoCl isomer induces a shortening of the micellar contour length. This reduction in contour length affects the viscosity, as we will discuss in the next paragraphs. The *trans–cis* transition does not induce a change in micellar morphology; therefore, no significant variation is observed in the hydrodynamic diameter or viscosity, indicating that tubular micelles remain interacting with each other.

Molecular dynamics simulations allow us to analyze the local spatial arrangement of particles, molecules, or chemical groups relative to a specific reference. In this case, we calculated the density number distribution of *trans*- and *cis*-AzoCH_3_ molecules relative to the center of the micelle. As a reference point, we selected the terminal methyl group (CH_3_) at the end of the hydrocarbon tail of each CTAB molecule.

Figure 9 shows the number density distributions of *cis*- and *trans*-AzoCH_3_ and *cis*- and *trans*-AzoCl in CTAB/NaSal systems. To characterize the spatial distribution of these molecules within the micelles, the centers of mass of the –NH_2_ group and the methylated or chloro-substituted aromatic ring were used as a representation of the most hydrophilic and hydrophobic regions, respectively. Distances were measured from these points to the terminal methyl group of the CTAB alkyl chain, taken as the reference position.

**Figure 9:**
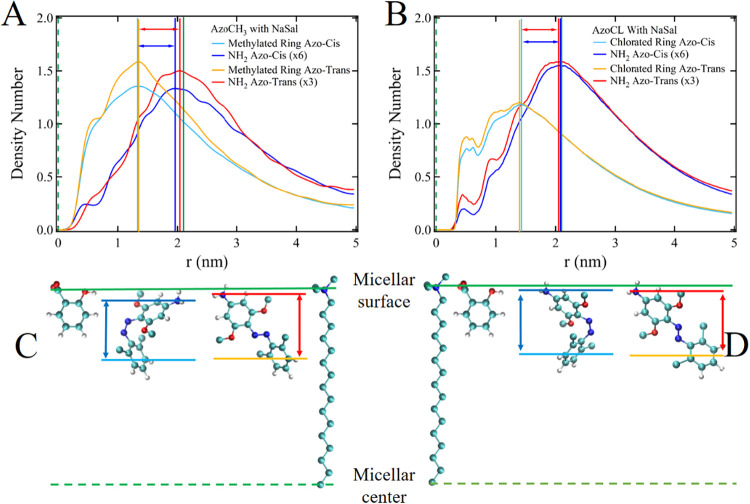
Density number distributions of the Azo functional groups (−NH_2_, chlorated aromatic ring and methylated aromatic ring) measured from the center of the micelle in (A) AzoCH_3_/CTAB/NaSal, and (B) AzoCl/CTAB/NaSal. Panels (C, D) provide schematic representations of the distances from the NH_2_ group and the methylated or chlorated aromatic ring to the micelle center, as well as the distance between them, illustrating their average proximity to the micelle surface.

Figure 9A presents the behavior of these distances in the CTAB/NaSal system (tubular micelles) for both isomers of AzoCH_3_. The distance from –NH_2_ group and the methylated ring is slightly greater for the *trans* isomer, indicating a closer surface position (Figure 9C), in both cases with the –NH_2_ group located near the micellar surface. This observation is consistent with the UV–Vis spectral analysis and with visual inspection of the simulation snapshots, which indicate that the AzoCH_3_ molecule preferentially localizes at the micellar surface, regardless of the isomer.

A similar result is observed for AzoCl in both isomers, as shown in Figure 9B, where distances between –NH_2_ group and the chloro-substituted aromatic ring are practically identical for both isomers. In this system, for the *cis* isomer the –NH_2_ group is located closer to the micellar surface compared to that in AzoCH_3_ as Figure 9C,D shows.

Additionally, the effective micellar radius of the tubular micelles was approximately 2.1 nm, estimated as the distance between the terminal methyl group (−CH_3_) and the nitrogen atom of the CTAB headgroup. This value is slightly higher than that obtained from TEM images (diameter ≈ 3.0 nm), which may be attributed to contrast enhancement and apparent size overestimation arising from phosphotungstic acid staining and drying effects during sample preparation.^40^ A schematic representation of this analysis for the *trans* and *cis* isomers of AzoCH_3_ and AzoCl is shown in Figure 9C,D, respectively, illustrating their distribution between the micellar center and the surface. The same color code used in the number-density plots (Figure 9A,B) was adopted to indicate the corresponding distances (Figure 9C,D).

The preferential localization of both azobenzene derivatives near the micellar interface obtained from the simulations is fully consistent with the conclusions derived from UV–Vis spectroscopy and thermal isomerization kinetics. Thus, independent experimental and computational approaches converge toward the same structural interpretation regarding azobenzene incorporation into the CTAB/NaSal assemblies.

Finally, based on the simulation analysis, we find that both isomers preferentially localize at the micellar surface for each Azo derivative. The conformational change of Azo upon photoisomerization modifies the distance between neighboring CTAB molecules and, consequently, the packing parameter, leading to a reduction in the contour length of the tubular micelles. Nevertheless, the system remains within the semidilute regime. Consistent with the snapshots shown in Figure 8, the tubular morphology is preserved after irradiation, although with shorter micellar structures. These results are in agreement with the morphology observed in TEM images and with the hydrodynamic diameter measurements obtained for both isomers in both systems.

After studying the microscopic and mesoscopic characteristics, we now proceed to analyze the macroscopic behavior. Through viscosity measurements, it is possible to explore the structural changes and verify whether the system preserves the characteristic behavior of a micellar system in the semidilute regime. Figure 10 shows the viscosity behavior of the four systems, in which the NaSal/CTAB ratio was varied (0.4 and 1) at a fixed CTAB concentration of 0.05 M, using both Azo concentrations of 0.0005 and 0.01 M. The red curve in all figures corresponds to the viscosity of the CTAB/NaSal system at the respective NaSal/CTAB ratios, used as reference.

**Figure 10:**
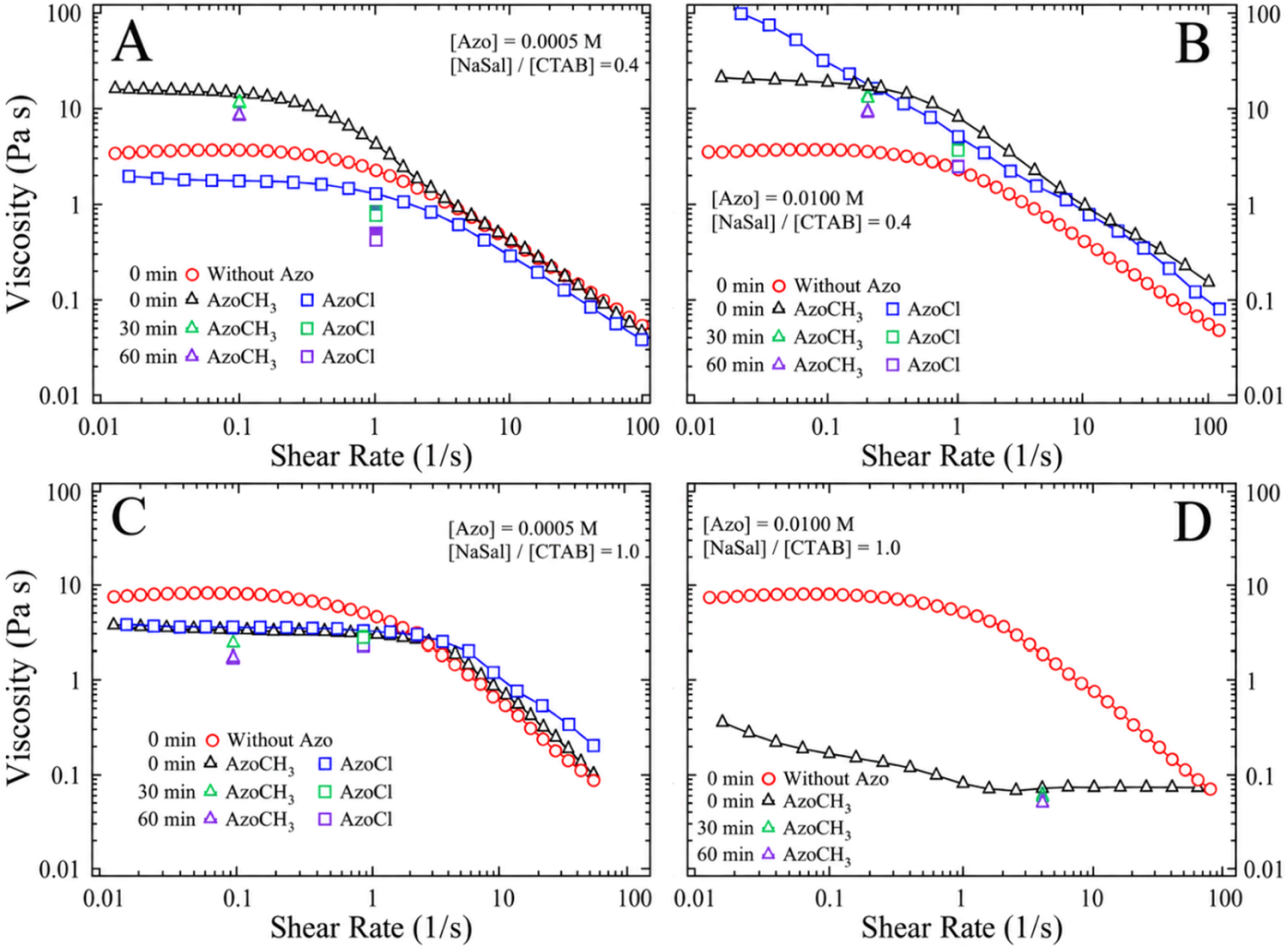
Viscosity for system without and with irradiation to different times (30 and 60 min) at pH 11: (A) [NaSal]/[CTAB] = 0.4 with concentration 0.0005 M of AzoCH_3_ and AzoCl, (B) [NaSal]/[CTAB] = 0.4 with concentration 0.01 M of AzoCH_3_ and AzoCl, (C) [NaSal]/[CTAB] = 1 with concentration 0.0005 M of AzoCH_3_ and AzoCl and (D) [NaSal]/[CTAB] = 1 with concentration 0.01 M of AzoCH_3_ and AzoCl.

Figure 10A,B show the viscosity behavior of the micellar systems at NaSal/CTAB ratios of 0.4, using AzoCH_3_ and AzoCl concentrations of 0.0005 and 0.0100 M, respectively. Measurements without irradiation were performed over a range of shear rates, whereas the complexity of measuring under irradiation only was performed at a single fixed shear rate to reduce acquisition time and minimize potential measurement errors associated with structural transitions. At low shear rates, nonirradiated samples exhibit Newtonian behavior, except for the system containing 0.0100 M AzoCl, which shows the highest viscosity. At low concentrations (Figure 10A), AzoCH_3_ increases the viscosity compared to the reference system, whereas AzoCl leads to a decrease. These trends are consistent with the corresponding behavior in hydrodynamic radius observed for the AzoCH_3_ and AzoCl systems, respectively. At higher shear rates, all samples display shear-thinning behavior, indicative of the presence of tubular micelles that align along the flow direction. In this regime, the viscosity follows a power law (η ≈ γ̇^(−α)^) with a slope α ≈ 0.8–1.0. Such scaling is characteristic of wormlike micellar systems in which micellar breakage occurs on time scales shorter than reptation, leading to stress relaxation dominated by reversible scission. This behavior is consistent with the nonlinear response of viscoelastic fluids described by the Maxwell model.^38^ This interpretation agrees with the structures obtained from simulations, where a tubular morphology is observed in the *trans*-Azo systems (Figure 8A–C). In the AzoCl system (Figure 8C,D), the tubular micelles exhibit greater contour length, which leads to an increase in viscosity, as shown in Figure 10B.

When the systems are irradiated, a slight decrease in viscosity is observed at the selected shear rate. This reduction is attributed to a decrease in the contour length of the micelles, consistent with simulation results showing shortening of tubular micelles and, in some cases, the formation of spherical micelles (Figure 8). Similar trends are observed in TEM images after irradiation, as well as a slight decrease in the hydrodynamic diameter measurements. These findings indicate that the system remains within the semidilute regime, where collective hydrodynamic interactions are preserved.

Figure 10C,D show the viscosities of the system at NaSal/CTAB ratios of 1.0, and again using AzoCH_3_ and AzoCl concentrations of 0.0005 and 0.0100 M, respectively. In Figure 10C, no significant differences in viscosity values are observed among the systems, and despite a slight reduction of viscosity related with the non-Azo system, all exhibit similar behavior, showing shear-thinning at high shear rates. However, as the Azo concentration increases, the viscosity behavior changes markedly. For AzoCH_3_ (Figure 10D), the viscosity significantly decreases, the slope of the shear-thinning region becomes less pronounced, and Newtonian behavior is observed at high shear rates. This structural change is likely induced by the high AzoCH_3_ concentration. Since the NaSal/CTAB ratio is 1, charge screening favors micellar growth; however, this effect is disrupted by the presence of AzoCH_3_, which modifies the packing parameter toward a more conical geometry. In contrast, phase separation is observed when 0.0100 M AzoCl is used, and we did not perform rheological measurements.

With the results from the rheological measurements, it can be concluded that the Azo transition induces a structural change; however, it is not strong enough to produce a marked morphological transition, likely due to the relatively small size of the Azo molecule and its high molecular hydrophobicity. To achieve morphological changes, further studies using azobenzene derivatives with larger molecular length could be explored. Subsequently, the incorporation of these azobenzene derivatives into CTAB/NaSal wormlike micellar systems may lead to more significant rheological responses. The combination with CTAB and NaSal wormlike micelles exhibits interesting behavior that appears to be strongly influenced by the electronic nature of the substituents, which can modulate the intermolecular interactions and, consequently, the viscoelastic properties of the self-assembled structures.

## Conclusions

The photoresponsive behavior of CTAB/NaSal micellar systems incorporating azobenzene derivatives (AzoCH_3_ and AzoCl) was systematically investigated by combining structural, dynamic, and kinetic analyses. Under basic conditions (pH 11), the addition of azobenzene molecules to CTAB/NaSal solutions leads to the formation of well-defined wormlike micelles (WLMs), as confirmed by TEM and molecular dynamics simulations. DLS measurements indicate that the systems lie within the semidilute regime, where collective hydrodynamic interactions govern the dynamics, consistent with the viscosity results. Therefore, the rheological behavior is primarily dictated by these collective effects. Under high shear conditions, a shear-thinning behavior is observed, attributed to the alignment of tubular micelles along the flow direction. UV irradiation at 360 nm induces *trans–cis* photoisomerization of the azobenzene moieties, resulting in a pronounced disruption of the WLM architecture. TEM observations reveal partial collapse and fragmentation of the tubular structures upon irradiation, demonstrating that azobenzene isomerization effectively modulates the local micellar structure while preserving the overall morphology. This is consistent with simulation results, which show a reduction in the contour length of the tubular micelles. Despite these substantial structural rearrangements, DLS results show no significant change in the characteristic hydrodynamic correlation length, indicating that the semidilute concentration regime is preserved. Similarly, the viscosity measured at a fixed shear rate shows a slight decrease, indicating a reduction in micellar contour length while preserving the tubular micellar morphology.

One of the most important findings of this work is that photoisomerization induces significant local structural modifications while preserving the semidilute wormlike micellar regime. This demonstrates that molecular-scale structural changes can be decoupled from the collective hydrodynamic organization of the network.

This finding highlights a clear decoupling between local micellar structure, mesoscopic organization (as reflected by DLS measurements), and macroscopic collective dynamics (as evidenced by viscosity). Thermal *cis*-to-*trans* isomerization kinetics monitored by UV–vis spectroscopy follow monoexponential behavior, confirming reversible photoresponsiveness of the azobenzene derivatives within the micellar environment. The preservation of semidilute behavior during repeated structural transitions underscores the robustness of the micellar network against molecular-level perturbations.

Overall, this study demonstrates that light-induced molecular transformations can be used to reversibly tune micellar morphology without altering the underlying concentration regime. These results provide fundamental insight into the hierarchical organization of photoresponsive micellar systems and point toward potential applications in stimuli-responsive soft materials where controlled structural modulation is desired without compromising collective solution properties. To induce more pronounced morphological transitions, future studies could explore azobenzene derivatives with greater molecular length.

## Supplementary Material



## Notes

Additional UV–vis spectroscopic data of azobenzene photoisomerization and thermal *cis–trans* relaxation experiments; recycling studies demonstrating photoswitching reversibility; comparative spectroscopic characterization in water and CTAB micelles; molecular dynamics simulations of CTAB/azobenzene systems without NaSal; DLS measurements before and after irradiation; and concentration-dependent DLS analyses of AzoCH_3_ and AzoCl in CTAB/NaSal wormlike micellar systems (PDF)
