# Supplementary material for: Multiscale Analysis of the Impact of the Isomerization of 4‑Amine Tetraortho-Azobenzenes on the Structure and Dynamics of Tubular Micelles
Source: Langmuir. 2026 Jun 18;42(25):18523–34. doi: 10.1021/acs.langmuir.6c02376 (PMC13325857; doi:10.1021/acs.langmuir.6c02376)
Supplement: Supplementary file 1 [file la6c02376_si_001.pdf]

Supplementary Information

Multiscale analysis of the impact of the isomerization of 4-amine tetraortho-azobenzenes on the structure and dynamics of tubular micelles

Alberto S. Luviano[1], Natalia Rincón-Londoño[2,*], Sandra Ramírez-Rave[3,*], Jesús Gracia-Mora[3], Maria Josefa Bernad-Bernad[3], Anatoly K. Yatsimirsky[3]

[1] Departamento de Ingenierías Química, Electrónica y Biomédica, División de Ciencias e Ingeniería, Universidad de Guanajuato, Campus León, León, Guanajuato 37150, México.

[2] Departamento de Ingeniería Física, División de Ciencias e Ingenierías, Universidad de Guanajuato, León, Guanajuato 37150, México.

[3] Facultad de Química, Universidad Nacional Autónoma de México, Ciudad Universitaria, Coyoacán, Ciudad de México 04510, México.

Corresponding authors: Natalia Rincón-Londoño natalia.rincon@ugto.mx and Sandra Ramírez-Rave sandraramirez@quimica.unam.mx.

Table of content

Figure SI1. $AzoCH_3$ CTAB/NaSal *cis* to *trans* isomerization experiment recycling the same solution after 7 days……………………………………………………….…………………..S2

Figure SI2. Spectral change observed in $AzoCH_3$ in water and CTAB micelles………………..S2

Figure SI3. Molecular dynamics simulations of systems without NaSal………………………S3

Figure SI4. DLS measurements for $AzoCH_3$/CTAB/NaSal system after and before irradiation at 360nm……………………………………………………………………………………S3

Figure SI6. DLS measurements of AzoCl at additional concentrations…………………………S4

Figure SI5. DLS measurements of AzoCH3 at additional concentrations………………………S4

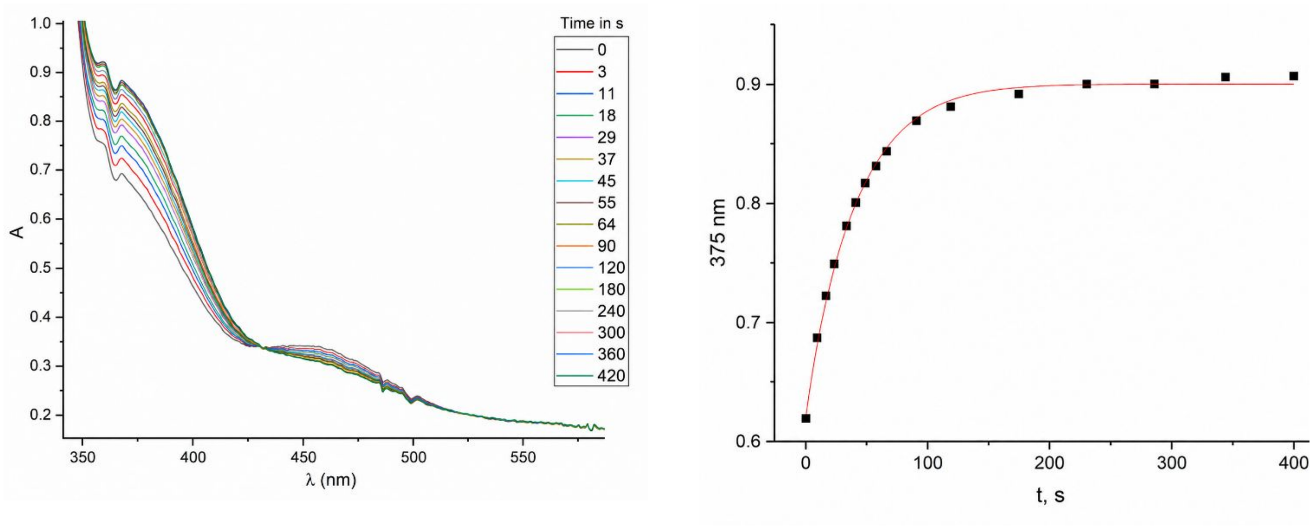

Figure SI1. $AzoCH_3$ CTAB/NaSal *cis* to *trans* isomerization experiment recycling the same solution after 7 days. ([NaSal]/[CTAB] = 0.4, pH 11 ($k = 2x10^{-2}\ s^{-1}$).

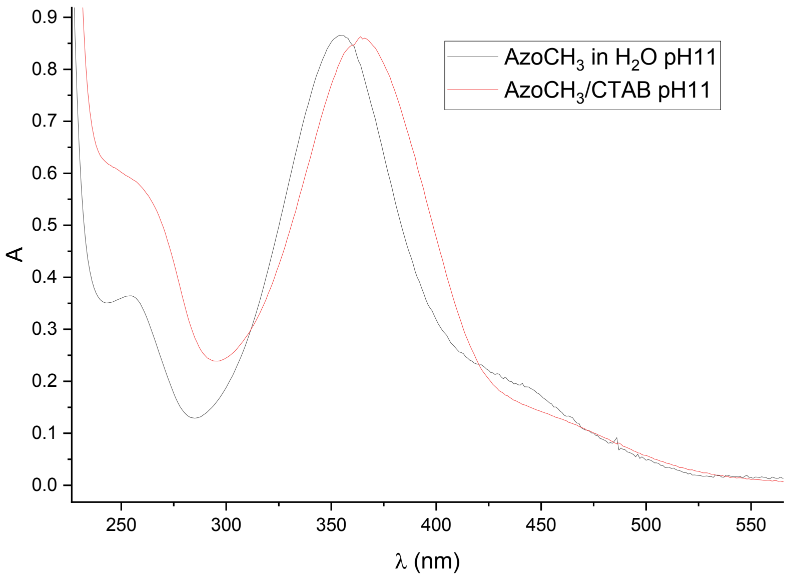

Figure SI2. Spectral change observed in $AzoCH_3$ in water and CTAB micelles.

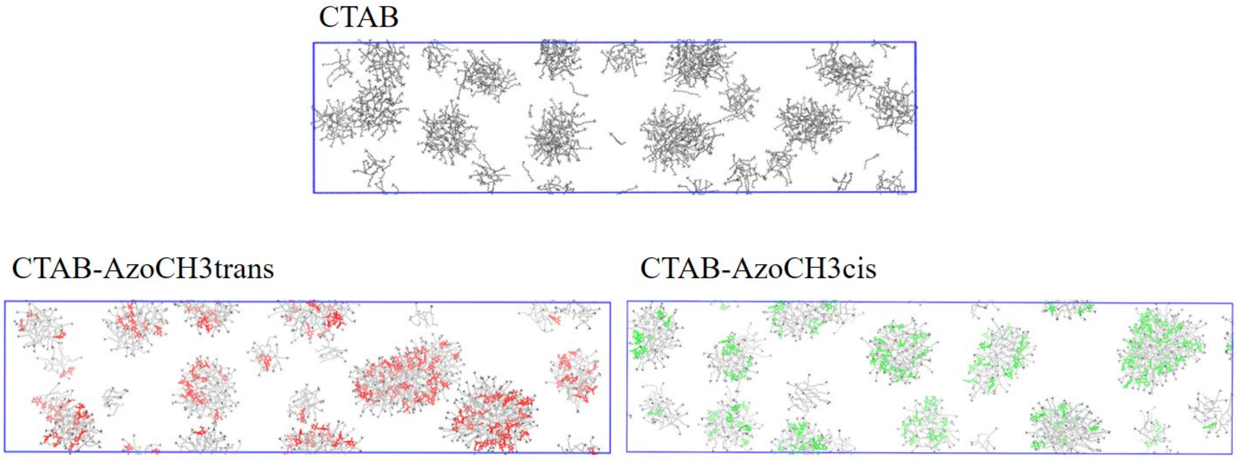

Figure SI3. Molecular dynamics simulations of systems without NaSal. Average radius of gyration of micelles remain around the same values, and no grew of micelles are observed. The average micelle diameters are 4.37 nm for CTAB, 4.64 nm for trans-$AzoCH_3$/CTAB, and 4.59 nm for cis-$AzoCH_3$/CTAB. All simulations were performed in a simulation box with dimensions of $10 \times 10 \times 40$ nm^3.

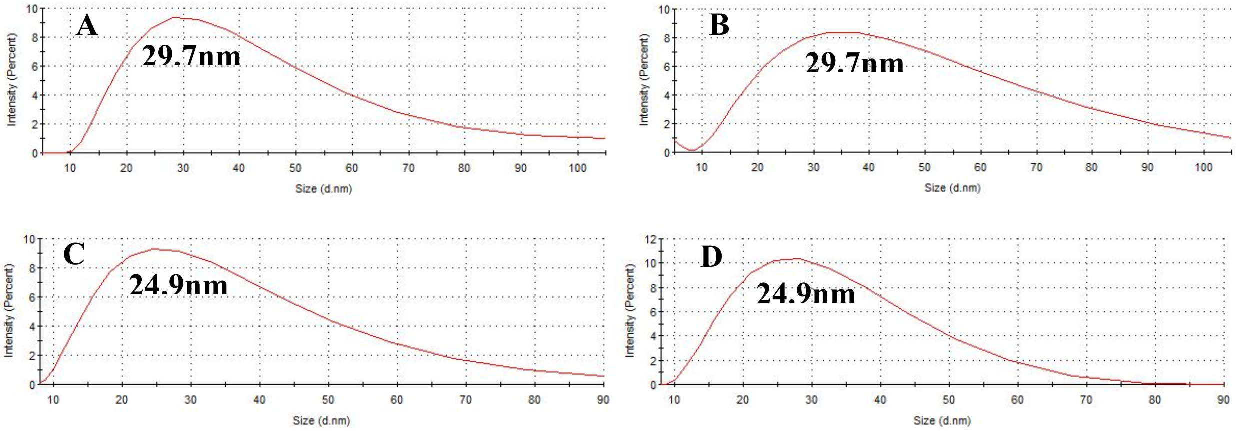

Figure SI4. DLS measurements for $AzoCH_3$/CTAB/NaSal system (**A**) after and (**B**) before irradiation at 360nm. These measurements were also taken for AzoCl/CTAB/NaSal (**C**) after and (**D**) before irradiation. ([NaSal]/[CTAB] = 0.4, pH 11.

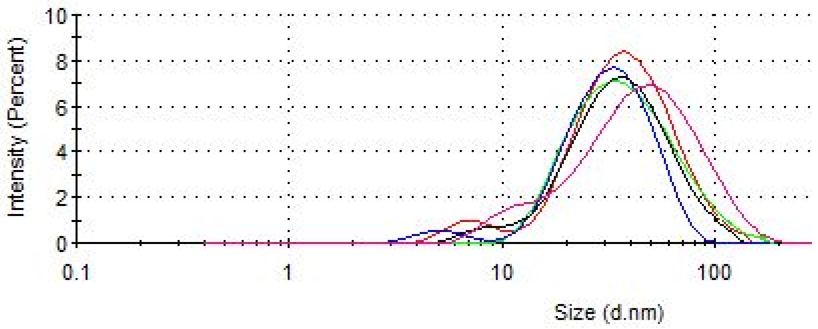

Figure SI5. DLS measurements of AzoCH3 at additional concentrations. -- CTAB/NaSal AzoCH$_3$ 0 M (25nm), -- CTAB/NaSal AzoCH$_3$ 2.5x10^{-5}M (28.9nm), -- CTAB/NaSal AzoCH$_3$ 5x10^{-5}M (29nm), -- CTAB/NaSal AzoCH$_3$ 5x10^{-4}M (32.1nm), -- CTAB/NaSal AzoCH$_3$ 5x10^{-3}M (35.3nm). ([NaSal]/[CTAB] = 0.4, pH 11.

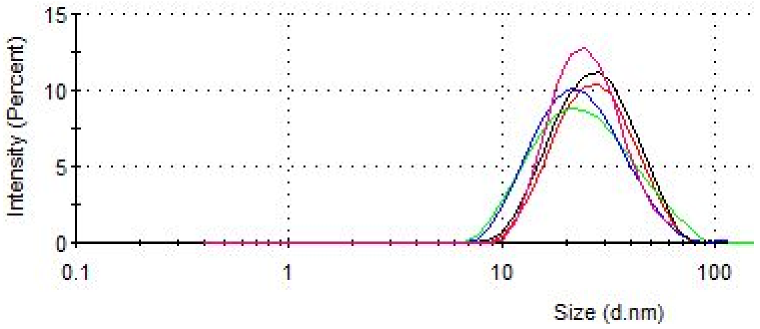

Figure SI6. DLS measurements of AzoCl at additional concentrations. -- CTAB/NaSal AzoCH$_3$ 0 M (24.1nm), -- CTAB/NaSal AzoCH$_3$ 2.5x10^{-5}M (24.5nm), -- CTAB/NaSal AzoCH$_3$ 5x10^{-5}M (25nm), -- CTAB/NaSal AzoCH$_3$ 5x10^{-4}M (25.1nm), -- CTAB/NaSal AzoCH$_3$ 5x10^{-3}M (24.9nm). ([NaSal]/[CTAB] = 0.4, pH 11.
